# In Vitro Antidiabetic Activity and Mechanism of Action of Methanolic Extract of *Opuntia stricta* Cladodes

**DOI:** 10.1155/bmri/9325356

**Published:** 2026-05-11

**Authors:** Martin Kampamba, Christian Chinyere Ezeala, Kadango Zombe, Angela Gono Bwalya

**Affiliations:** ^1^ Department of Pharmacy, School of Health Sciences, University of Zambia, Lusaka, Zambia, unza.zm; ^2^ College of Health, Agriculture, and Natural Sciences, Africa University, Mutare, Zimbabwe, africau.edu; ^3^ Department of Chemistry, School of Pure and Applied Sciences, University of Zambia, Lusaka, Zambia, unza.zm

## Abstract

**Background and Aims:**

Type 2 diabetes mellitus (T2DM) is characterized by insulin resistance and *β*‐cell dysfunction, leading to chronic hyperglycemia. Although *Opuntia* species have demonstrated antidiabetic potential, evidence on the in vitro mechanisms of *Opuntia stricta* cladodes (OSCs) remains limited. This study investigated the antidiabetic activity and mechanism of action of methanolic extracts of OSC using multiple cell‐based assays.

**Methods:**

Cladodes were collected in Zambia, shade‐dried, powdered, and extracted with methanol. Cytotoxicity and antidiabetic effects were assessed using C3A hepatocytes, L6 myoblasts, Caco‐2 epithelial cells, and INS‐1 *β*‐cells. Assays included cell viability, glucose utilization, glucose uptake, and *β*‐cell proliferation. Data were analyzed using Student′s *t*‐test and one‐way ANOVA with Tukey′s post hoc test (*p* < 0.05).

**Results:**

The OSC extract showed good tolerability, with cell viability > 80% across all cell lines. In C3A hepatocytes, glucose utilization increased significantly at 6.25–100 *μ*g/mL (*p* < 0.001), with the highest effect at 100 *μ*g/mL (90.1*%* ± 0.3*%*) compared to untreated (86.3*%* ± 0.4*%*). Glucose uptake was also significantly enhanced at 100 *μ*g/mL compared to untreated (51.2*%* ± 1.0*%* vs. 43.4*%* ± 2.5*%*, *p* < 0.001) but without a significant difference from insulin (50.2*%* ± 3.6*%*, *p* = 0.984). After 24 h in L6 myoblasts, treatment at 100 *μ*g/mL modestly but significantly increased glucose utilization compared with untreated controls (95.06*%* ± 0.44*%* vs. 93.5*%* ± 0.59*%*, *p* < 0.005). Uptake also increased significantly at 100 *μ*g/mL when compared to untreated (53.7*%* ± 1.7*%* vs. 48.5*%* ± 2.6*%*, *p* < 0.005) but was not significantly different from insulin (53.7*%* ± 1.7*%* vs. 54.3*%* ± 2.0*%*, *p* = 0.999). In Caco‐2 cells, glucose uptake decreased compared with untreated controls at 6.25 *μ*g/mL (57.5*%* ± 4.9*%* vs. 64.6*%* ± 3.6*%*, *p* < 0.05) and 100 *μ*g/mL (50.8*%* ± 9.4*%* vs. 64.6*%* ± 3.6*%*, *p* < 0.001). In INS‐1 *β*‐cells, proliferation increased at 125 *μ*g/mL after 24 h compared to untreated (111.8*%* ± 2.2*%* vs. 100.0*%* ± 3.8*%*, *p* < 0.001), exceeding 10% fetal bovine serum (FBS) (97.8*%* ± 4.1*%*) but declined significantly at 48 and 72 h (*p* < 0.05).

**Conclusion:**

OSC extracts enhanced glucose utilization and uptake in C3A hepatocytes and L6 myoblasts to levels similar to insulin, reduced intestinal glucose uptake in Caco‐2 cells relative to untreated controls, and transiently stimulated *β*‐cell proliferation beyond that of 10% FBS. These findings highlight OSC as a promising source of antidiabetic bioactives, warranting further molecular and in vivo studies.

## 1. Background

The prevalence of Type 2 diabetes mellitus (T2DM) globally has reached epidemic proportions [1]. According to the International Diabetes Federation (IDF) Diabetes Atlas (2025), it is estimated that 11.1% of adults aged 20–79 years are living with diabetes. Additionally, it is projected that this number may rise to 1 in 8 adults by 2050 [2, 3]. Literature has shown that about 25 million adults in Africa are living with diabetes mellitus (DM), with nearly 70% undiagnosed. This makes Africa a region with the highest prevalence of undiagnosed DM [4–6]. In Zambia, a lower‐middle‐income country in sub‐Saharan Africa, there is a scarcity of national data on the prevalence of DM and its associated factors. The prevalence of diabetes in Zambia is around 4.2%. However, some national estimates put this figure as high as 10.3% [7].

Insulin resistance serves as the key pathophysiological link between obesity and T2DM. It refers to a diminished responsiveness of insulin′s target tissues to normal concentrations of circulating insulin [8, 9]. This metabolic state, frequently associated with dysfunction of the pancreas, results in chronic hyperglycemia and subsequently T2DM [9]. Uncontrolled hyperglycemia and sustained insulin resistance are usually major drivers of serious DM complications, in particular diabetic nephropathy, neuropathy, and retinopathy. Furthermore, these increase the risk of cardiovascular morbidity and mortality [10, 11]. The growing body of evidence has revealed that T2DM is also a risk factor for a number of neurodegenerative disorders, such as Alzheimer′s disease and Parkinson′s disease [12, 13].

Nutritional interventions have long served as a keystone in preventing and managing T2DM. Diets, particularly the Mediterranean diet, have been documented to enhance glycemic control [14]. One increasing example is the utilization of products derived from cactus, such as those from the prickly pear (PP) (*Opuntia* spp.) cladodes. *Opuntia* spp. have drawn interest for their potential role in controlling the levels of blood glucose and improving metabolic health [15, 16]. Studies have revealed that the consumption of cladodes of *Opuntia* spp. produces acute hypoglycemic effects [17]. One study in South Korea showed that *Opuntia ficus-indica* var. *saboten* cladode powder extract displayed in vitro hypoglycemic activity in rats with L6 myoblasts [18]. Additionally, another study revealed that cladode supplementation in obese rats fed a high‐sucrose diet enhanced metabolic health, decreasing triglycerides, total cholesterol, and gastric inhibitory polypeptide (GIP) levels while improving glycemic control and insulin sensitivity [19].

The antidiabetic properties and mechanisms of action of *Opuntia* species have been evaluated using in vitro studies [20]. However, the exact mechanisms responsible for these hypoglycemic effects remain incompletely elucidated [21]. Remarkably, far fewer studies have explicitly evaluated the in vitro mechanisms of action of *Opuntia stricta* cladodes (OSCs), with a lot of research focusing on *Opuntia ficus-indica* and *Opuntia dillenii* [22–26]. Therefore, the current study is aimed at evaluating the in vitro antidiabetic activity and mechanism of action of OSC methanolic extract.

## 2. Materials and Methods

### 2.1. Plant Collection and Identification

Cladodes of *Opuntia stricta* measuring 2–5 cm in length (12‐day‐old) and 10–20 cm in length (20‐day‐old) were collected in October from Chibombo District, Chief Mungule, Zambia (altitude: ~1283 m [4290 ft] above sea level). Botanical identification was confirmed by taxonomists from the Department of Biological Sciences, University of Zambia. All laboratory and experimental work was performed at the Department of Biochemistry, University of Nelson Mandela, South Africa.

### 2.2. Extraction Preparation

Cladodes aged 12 days (mean weight: 32 g, standard deviation [SD] ±10 g) and 20 days (mean weight: 70 g, SD ±20 g) were washed with sterile water and disinfected with 10% sodium hypochlorite solution. The two age groups were pooled prior to extraction, as previous studies have shown that the phytochemical composition and bioactive properties of *Opuntia* cladodes vary with maturity, with young to medium‐aged cladodes often exhibiting higher levels of bioactive compounds than older cladodes [27]. Thorns were removed using a stainless‐steel knife, and the cladodes were cut into 2 × 2‐cm^2^ pieces. The material was shade‐dried at room temperature for 3 weeks and ground in an electric mill to obtain particles < 4 mm. For extraction, 100 g of powdered material was combined with 330 mL of ethanol, methanol, or acetone and agitated for 15 min every 4 h over 48 h. The mixtures were filtered through Whatman No. 1 paper, and the filtrates were concentrated to dryness under reduced pressure using a rotary evaporator for 15 min [27]. Percentage yield was calculated as follows:

Weight of crude extractWeight of dry plant material×100,Methanol%yield:11 g100 g×10011=%,Ethanol%yield:9 g100 g×1009=%,Acetone%yield:5 g100 g×1005=%.

The extracts were stored and refrigerated at 6°C–8°C for further use [27, 28]. Methanol demonstrated the highest percentage yield and proved to be the most suitable solvent for the broad‐spectrum extraction of phytochemicals from OSC. Consequently, it was selected for subsequent analyses, as presented in Section 3.1.

Crude OSC extract was weighed into 50 mg aliquots, transferred into 1.0‐mL microcentrifuge tubes, and then dissolved in DMSO to prepare a concentrated stock solution. The stock solution was subsequently diluted in complete culture medium to obtain working concentrations ranging from 6.25 to 125 *μ*g/mL for the cell‐based assays.

### 2.3. Qualitative Phytochemical Screening of OSC Extracts

Qualitative phytochemical screening of the acetone, methanol, and ethanol extracts of OSCs was performed using standard phytochemical methods to detect major classes of secondary metabolites. The extracts were evaluated for the presence of anthraquinones, tannins, saponins, flavonoids, terpenoids, glycosides, phenols, and reducing sugars following previously described procedures [29, 30]. The specific tests employed and their corresponding observations are summarized in Table 1.

**Table 1:** Qualitative phytochemical screening of *Opuntia stricta* cladode extracts.

Phytochemical	Test method	Procedure	Observation (positive result)
Anthraquinones	Borntrager′s test	Benzene was added to the powdered sample, soaked, and filtered. Ammonia solution was added to the filtrate and shaken.	Pink, violet, or red color in the ammonia layer indicated the presence of anthraquinones.
Tannins	Bromine water test	Bromine water was added to the aqueous extract.	Decolorization of bromine water indicated the presence of tannins.
Saponins	Froth′s test	Distilled water was added to the extract and shaken vigorously. Olive oil was then added, and the mixture was shaken again.	Stable froth or foam formation indicated the presence of saponins.
Flavonoids	Shinoda′s test	Magnesium ribbon and concentrated HCl were added to the extract.	Development of a pink or red color indicated the presence of flavonoids.
Flavonoids	Alkaline reagent test	Sodium hydroxide solution was added to the extract, followed by dilute acid.	Formation of a yellow color that disappeared upon acid addition indicated flavonoids.
Terpenoids	Salkowski′s test	Chloroform was added to the extract, evaporated, and treated with concentrated sulfuric acid.	Formation of a grey color indicated the presence of terpenoids
Glycosides	Liebermann′s test	Acetic acid and chloroform were added to the extract, followed by concentrated sulfuric acid.	Formation of a green color indicated the presence of steroidal glycosides.
Glycosides	Keller–Kiliani′s test	Glacial acetic acid containing ferric chloride was added to the extract, followed by sulfuric acid.	Formation of a brown ring between layers indicated cardiac glycosides.
Glycosides	Salkowski′s test	Concentrated sulfuric acid was added to the extract.	Formation of a reddish‐brown color indicated steroidal aglycones.
Phenols	Ferric chloride test	Ferric chloride solution was added to the extract.	Development of red, green, or purple color indicated phenolic compounds.
Reducing sugars	Benedict′s test	Extract filtrate was mixed with Benedict′s reagent and heated in a water bath.	Formation of a brick‐red precipitate indicated reducing sugars.

### 2.4. Cell Lines, Media, Reagents, and Assay Kits

Accurate quantification of cell viability and proliferation is essential when performing multiple in vitro assays, as cells can respond variably to external stimuli. In this study, cell viability was assessed to ensure that any observed changes in glucose uptake and utilization, as well as in *β*‐cell proliferation, were not confounded by alterations in cell number or viability. Total nuclei were quantified using Hoechst 33342, a membrane‐permeant dye that binds the minor groove of double‐stranded DNA (with enhanced fluorescence in A–T‐rich regions) and, upon excitation at 350 nm, emits blue fluorescence at 461 nm, enabling whole‐population nuclear counts [31]. The cell viability assessment was undertaken for all cell lines employed, ensuring that the concentrations of the extracts and fractions tested did not exert detrimental effects on cell survival. For the evaluation of glucose utilization and uptake in hepatic cells, the C3A subclone of HepG2 cells was utilized [32, 33]. Glucose utilization and uptake in skeletal muscle cells were measured using rat L6 myoblasts, following established methodologies [34–36]. The proliferation of pancreatic *β*‐cells was assessed using rat insulin‐secreting INS‐1 cells [37]. Furthermore, glucose absorption and metabolism in intestinal cells were investigated using Caco‐2 cells, in accordance with previously published protocols [38].

The INS‐1 cells (*Rattus norvegicus*, male, pancreatic *β*‐cell; AddexBio, RRID:CVCL_0351), Caco‐2 cells (*Homo sapiens*, male, colon epithelium; American Type Culture Collection [ATCC] HTB‐37, RRID:CVCL_0025), and L6 myoblasts (*Rattus norvegicus*, male, skeletal muscle myoblast; RIKEN Cell Bank, RRID:CVCL_0385) were procured from Highveld Biological, South Africa, while the C3A subclone of HepG2 cells (*Homo sapiens*, female, hepatocytes, liver tissue; ATCC CRL‐10741, RRID:CVCL_1848) was sourced from the ATCC (Manassas, Virginia, United States). Authentication was verified by short tandem repeat (STR) profiling, showing a ≥ 95% match to the reference profile, and none of the cell lines have been reported as misidentified or contaminated in the Cellosaurus database. Routine mycoplasma testing using MycoAlert Mycoplasma Detection kit (Lonza, United States) was performed before each experiment run, confirming all cultures were free from mycoplasma contamination.

### 2.5. Maintenance of Cell Cultures

HepG2, L6 myoblast, INS‐1, and Caco‐2 cells were cultured at 37°C in a humidified incubator with 5% CO_2_. HepG2, L6, and Caco‐2 cells were maintained in antibiotic‐free Dulbecco′s Modified Eagle Medium (DMEM) supplemented with 10% fetal bovine serum (FBS), whereas INS‐1 cells were maintained in antibiotic‐free Roswell Park Memorial Institute (RPMI) 1640 medium with 10% FBS [39, 40].

### 2.6. Cell Viability Testing

To evaluate treatment‐related cytotoxicity, cell viability was assessed by nuclear staining and image‐based analysis. Cell viability results were interpreted using a tiered system consistent with the International Organization for Standardization (ISO) 10993‐5 guidance and peer‐reviewed literature, where viability ≥ 80% was considered noncytotoxic, 70%–79% as mild cytotoxicity, 50%–69% as moderate cytotoxicity, and< 50% as severe cytotoxicity (ISO 10993‐5:2009). After treatment, residual medium was aspirated, and cells were stained with Hoechst 33342 (5 *μ*g/mL in PBS containing Ca^2+^/Mg^2+^)—100 *μ*L per well for C3A hepatocytes, L6 myoblasts, and Caco‐2 cells and 50 *μ*L per well for INS‐1 cells—followed by a 30‐min incubation. Fluorescent micrographs were then acquired on an ImageXpress Micro XLS Widefield Microscope (Molecular Devices) using a 10× Plan Fluor objective and a DAPI filter cube. Images were analyzed in MetaXpress with the Multi‐Wavelength Cell Scoring module to obtain total nuclei counts, and viability was expressed as a percentage of the untreated control based on total cell number [31].

### 2.7. Determination of Glucose Utilization and Uptake in L6 Myoblast, C3A Hepatocytes, and Caco‐2 Cells

Glucose utilization and uptake were assessed in C3A hepatocytes, L6 myoblasts, and Caco‐2 cells as described by a previous study [38]. Cells were seeded in 96‐well plates at a density of 2 × 10^4^ cells/well in 100 *μ*L aliquots and allowed to attach overnight. Treatments were prepared in complete medium containing the test samples at final concentrations of 6.25, 12.5, 25, 50, and 100 *μ*g/mL, added to the cells, and incubated for 24 h. For glucose utilization, 10 *μ*L of spent culture medium was collected after the 24‐h treatment period, transferred to a new 96‐well plate, and stored at −20°C until analysis, representing the cumulative change in glucose concentration during the treatment period.

For glucose uptake, after the 24‐h treatment, the remaining medium was aspirated, and cells were washed with 100 *μ*L PBS. An incubation buffer (RPMI‐1640 diluted with PBS containing 0.1% BSA, adjusted to 8.5 mM glucose) was then added to the cells (25 *μ*L/well). C3A hepatocytes and L6 myoblasts received insulin as a positive control, whereas Caco‐2 cells were assessed without a positive control. Following a 4‐h incubation, 10 *μ*L of culture medium was collected for analysis, representing acute glucose uptake posttreatment.

Glucose concentration in all collected samples (utilization and uptake) was quantified using a freshly prepared glucose oxidase–peroxidase reagent containing 3 mM phenol, 0.4 mM 4‐aminoantipyrine, 0.25 mM EDTA, 2.5 U/mL horseradish peroxidase, and 1 mU/mL glucose oxidase from *Aspergillus niger* in 0.5 M PBS (pH 7.0). Aliquots (200 *μ*L) of the reagent were added to the samples and incubated for 15 min at room temperature to allow formation of a red quinoamine dye. Absorbance was measured at 510 nm using a BioTek PowerWave XS spectrophotometer (Winooski, Vermont, United States). Cell‐free wells containing incubation buffer and complete culture medium were used to prepare glucose standards.

### 2.8. The *β*‐Cell Proliferation Assay

The *β*‐cell proliferation assay was performed as previously described [41]. Cells (2 × 10^4^ cells/well, 100 *μ*L) were seeded into 96‐well plates and allowed to attach overnight. Treatments prepared in complete medium (RPMI‐1640 supplemented with 10% FBS) containing the test samples at concentrations of 31.25, 62.5, and 125 *μ*g/mL were applied for 24, 48, or 72 h. At each time point, the culture medium was aspirated and replaced with 50 *μ*L of staining solution containing bis‐benzamide H 33342 trihydrochloride (Hoechst 33342, 5 *μ*g/mL) in PBS with Ca^2+^ and Mg^2+^. Cells were incubated with the staining solution for 30 min to allow nuclear labeling, after which fluorescent micrographs were captured using an ImageXpress Micro XLS Widefield Microscope (Molecular Devices) equipped with a 10× Plan Fluor objective and DAPI filter cube. Hoechst staining was applied only during the imaging step to enable nuclear visualization and cell counting, and exposure times during image acquisition were minimized to reduce potential phototoxic effects. Images were analyzed using MetaXpress software with the Multi‐Wavelength Cell Scoring Application Module to determine total cell numbers.

### 2.9. Statistical Analysis

All analyses were performed in GraphPad Prism Version 8.0 (GraphPad Software, San Diego, California, United States). For the INS‐1 *β*‐cell proliferation assay, replicate wells for each treatment were compared with replicate control wells using a two‐tailed Student′s *t*‐test. Statistical significance was defined as *p* < 0.05 (with observed *p* values ranging from < 0.001 to < 0.05). For all other experiments, group differences were evaluated by one‐way analysis of variance (ANOVA) followed by Tukey′s post hoc multiple‐comparison test. Data were reported as mean ± standard deviation (SD), and values with *p* < 0.05 were considered statistically significant.

## 3. Results

### 3.1. Phytochemical Analysis of OSCs in Acetone, Ethanol, and Methanol Solvents

As presented in Table 2, acetone, ethanol, and methanol were used as extraction solvents for the qualitative phytochemical screening of OSC extracts. The results demonstrated differences in the phytochemical constituents recovered by each solvent, reflecting variations in their extraction efficiencies. Among the solvents evaluated, methanol exhibited the highest extraction efficiency, yielding comparatively greater amounts of reducing sugars, alkaloids, tannins, glycosides, and carbohydrates. Ethanol showed moderate extraction capability, particularly for alkaloids, terpenes, glycosides, flavonoids, and saponins. In contrast, acetone generally extracted fewer phytochemical constituents but showed relatively higher efficiency in recovering triterpenoids and sterols, which are typically more soluble in solvents of lower polarity. Furthermore, anthraquinones were not detected in any of the solvent extracts. Collectively, these findings indicate that methanol was the most effective solvent for the broad‐spectrum extraction of phytochemicals from OSCs and was therefore selected for subsequent analyses.

**Table 2:** Phytochemical analysis of *Opuntia stricta* cladodes in acetone, ethanol, and methanol.

Test	Type of solvent		
	Acetone	Ethanol	Methanol
Tannins	**+**	**++**	**+++**
Saponins	**−**	**++**	**++**
Flavonoids	**+**	**++**	**+++**
Glycosides	**−**	**++**	**+++**
Alkaloids	**+**	**+++**	**+++**
Terpenes	**+**	**++**	**++**
Sterols	**+++**	**++**	**++**
Reducing sugars	**++**	**+**	**+**
Carbohydrates	++	++	+++
Triterpenoids	**+++**	**++**	**++**
Anthraquinone	**−**	**−**	**−**

### 3.2. Cell Viability in L6 Myoblast and C3A Hepatocytes

**Figure 1:**
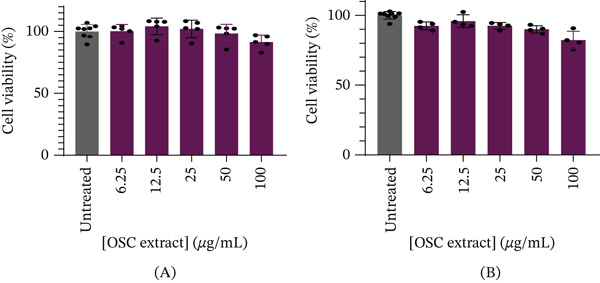
Cell viability (%). (A) L6 myoblast cells and (B) C3A cells after 24 h of exposure to treatment as indicated.

As shown in Figure 1A, no significant (all adjusted *p* > 0.05) cytotoxicity was observed in L6 myoblasts at any tested concentration (6.25–100 *μ*g/mL) when compared to the untreated control, indicating good tolerability in this cell line. The mean viability values (±SE) were 100.0*%* ± 5.63*%* (untreated), 100.2*%* ± 6.29*%* (6.25 *μ*g/mL), 104.1*%* ± 7.68*%* (12.5 *μ*g/mL), 102.1*%* ± 8.23*%* (25 *μ*g/mL), 98.34*%* ± 8.61*%* (50 *μ*g/mL), and 91.42*%* ± 6.50*%* (100 *μ*g/mL). In contrast, as shown in Figure 1B, multiple‐comparison testing demonstrated that C3A hepatocyte viability was significantly reduced at 6.25 *μ*g/mL (92.38*%* ± 2.77*%*, *p* = 0.0270), 25 *μ*g/mL (92.46*%* ± 2.37*%*, *p* = 0.0294), 50 *μ*g/mL (89.93*%* ± 2.63*%*, *p* = 0.0022), and 100 *μ*g/mL (82.20*%* ± 2.77*%*, *p* < 0.0001) compared with the untreated control (100*%* ± 2.71*%*). However, all values remained above the 80% viability threshold, indicating noncytotoxicity. No significant difference was observed between 12.5 *μ*g/mL (95.77*%* ± 4.58*%*, *p* = 0.4354) and the untreated control.

### 3.3. Glucose Utilization in L6 Myoblast and C3A Hepatocytes

**Figure 2:**
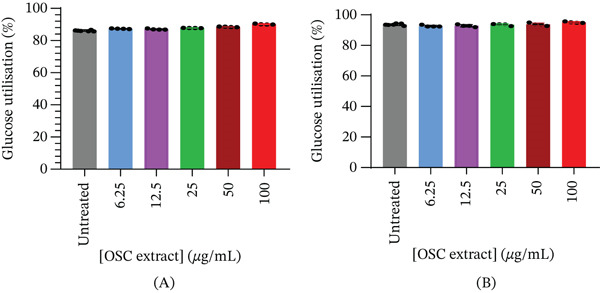
Glucose utilization (%) after 24 h of treatment in (A) L6 myoblasts and (B) C3A hepatocytes.

Glucose utilization measured after 24 h of treatment (Figure 2)A showed a modest but statistically significant increase in L6 myoblasts at 100 *μ*g/mL (95.06*%* ± 0.44*%*, *p* < 0.005) compared to untreated controls (93.51*%* ± 0.59*%*). No significant (all adjusted *p* > 0.05) differences were observed between the untreated control and concentrations of 6.25–50 *μ*g/mL. The mean glucose utilization values were 92.67*%* ± 0.55*%* (6.25 *μ*g/mL), 92.84*%* ± 0.77*%* (12.5 *μ*g/mL), 93.51*%* ± 0.62*%* (25 *μ*g/mL), and 93.76*%* ± 0.90*%* (50 *μ*g/mL). In C3A hepatocytes (Figure 2)B, glucose utilization was significantly (all adjusted *p* < 0.001) elevated at concentrations of 6.25–100 *μ*g/mL compared to the control, indicating a dose‐responsive metabolic activation. The mean glucose utilization values were 86.3*%* ± 0.4*%* (untreated), 87.31*%* ± 0.16*%* (6.25 *μ*g/mL), 87.04*%* ± 0.36*%* (12.5 *μ*g/mL), 87.78*%* ± 0.07*%* (25 *μ*g/mL), 88.50*%* ± 0.21*%* (50 *μ*g/mL), and 90.1*%* ± 0.3*%* (100 *μ*g/mL).

### 3.4. Glucose Uptake in L6 Myoblast and C3A Hepatocytes

**Figure 3:**
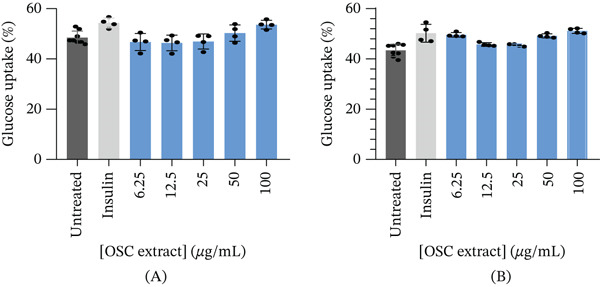
Glucose uptake (%) after 4 h in (A) L6 myoblasts and (B) C3A hepatocytes following a 24‐h treatment period.

Regarding glucose uptake in L6 myoblasts, assessed after 4 h of incubation following a 24‐h pretreatment (Figure 3)A, multiple‐comparison analysis showed a slight but significant increase at 100 *μ*g/mL (53.7*%* ± 1.7*%*) compared with the untreated control (48.5*%* ± 2.6*%*, *p* < 0.005). Insulin (100 ng/mL) also significantly increased glucose uptake (54.3*%* ± 2.0*%*) compared with 6.25 *μ*g/mL (46.7*%* ± 3.4*%*, *p* = 0.0101), 12.5 *μ*g/mL (46.4*%* ± 3.1*%*, *p* = 0.0065), and 25 *μ*g/mL (46.9*%* ± 3.0*%*, *p* = 0.0128). However, insulin did not differ significantly from the responses observed at 50 *μ*g/mL (50.3*%* ± 3.3*%*) or 100 *μ*g/mL (53.7*%* ± 1.7*%*). Multiple‐comparison analysis in C3A hepatocytes (Figure 3)B showed that OSC significantly increased glucose uptake at certain concentrations, with the highest response observed at 100 *μ*g/mL. Insulin (100 ng/mL) significantly increased glucose uptake (50.2*%* ± 3.6*%*) compared with the untreated control (43.4*%* ± 2.5*%*, *p* < 0.0001), as well as with 12.5 *μ*g/mL (45.8*%* ± 0.7*%*, *p* = 0.0357) and 25 *μ*g/mL (45.5*%* ± 0.5*%*, *p* = 0.0225). However, insulin did not differ significantly from the responses observed at 6.25 *μ*g/mL (49.6*%* ± 0.9*%*, *p* = 0.9986), 50 *μ*g/mL (49.2*%* ± 0.8*%*, *p* = 0.9842), or 100 *μ*g/mL (51.2*%* ± 1.0*%*, *p* = 0.9910).

### 3.5. Cell Viability in Caco‐2 Cells

**Figure 4:**
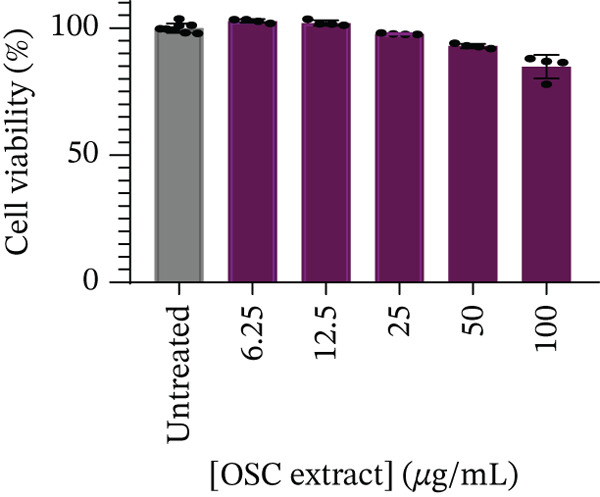
Cell viability (%) Caco‐2 cells after 24 h of exposure to treatment as indicated.

As shown in Figure 4, no significant cytotoxicity was observed in Caco‐2 cells at 6.25 *μ*g/mL (102.74*%* ± 0.71*%*, *p* = 0.3023), 12.5 *μ*g/mL (101.93*%* ± 1.03*%*, *p* = 0.6618), and 25 *μ*g/mL (97.67*%* ± 0.25*%*, *p* = 0.4690). However, significant reductions in viability were observed at 50 *μ*g/mL (92.88*%* ± 0.87*%*, *p* < 0.001) and 100 *μ*g/mL (84.77*%* ± 4.67*%*, *p* < 0.001) compared with the untreated control (100.00*%* ± 1.83*%*). Despite these reductions, all test concentrations produced viability values above the 80% threshold, indicating noncytotoxicity.

### 3.6. Glucose Utilization and Uptake in Caco‐2 Cells

**Figure 5:**
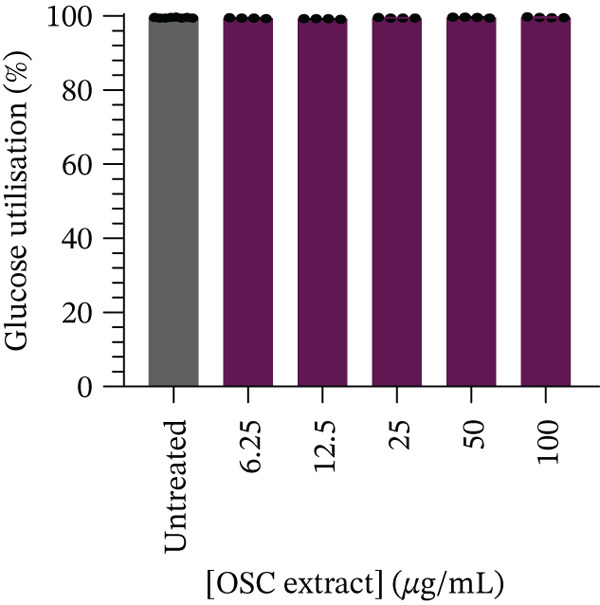
Glucose utilization (%) after 24 h of treatment in Caco‐2 cells.

**Figure 6:**
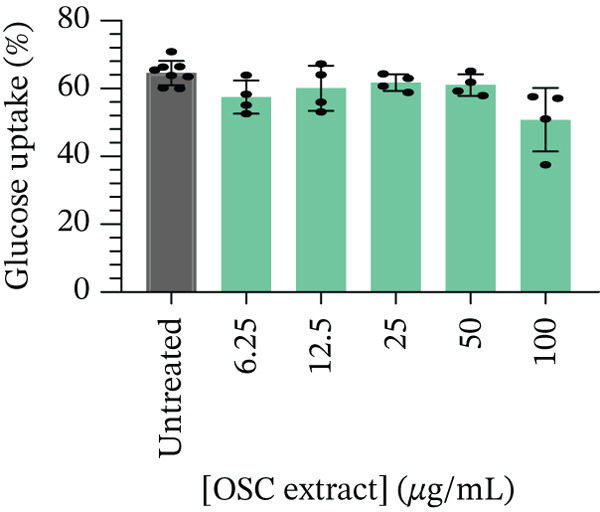
Glucose uptake (%) after 4 h of treatment in Caco‐2 cells.

After 24 h of treatment, glucose utilization analysis revealed that almost all glucose present in the culture medium had been consumed across all tested concentrations, indicating that the cells had entered a glucose‐depleted state by the end of the incubation (Figure 5). No significant changes in glucose utilization were observed at 6.25 *μ*g/mL (99.43*%* ± 0.12*%*, *p* = 0.5852), 12.5 *μ*g/mL (99.26*%* ± 0.07*%*, *p* = 0.1234), 25 *μ*g/mL (99.49*%* ± 0.07*%*, *p* = 0.9865), 50 *μ*g/mL (99.65*%* ± 0.12*%*, *p* = 0.4696), or 100 *μ*g/mL (99.62*%* ± 0.11*%*, *p* = 0.7380) when compared to the untreated control (99.53*%* ± 0.10*%*). Subsequent assessment of glucose uptake over a 4‐h period demonstrated a statistically significant reduction at 6.25 *μ*g/mL (57.47*%* ± 4.91*%*, *p* < 0.05) and 100 *μ*g/mL (50.80*%* ± 9.37*%*, *p* < 0.001) compared with untreated controls (64.55*%* ± 3.57*%*) (Figure 6). Although reductions were also observed at higher concentrations, 12.5 *μ*g/mL (60.09*%* ± 4.91*%*, *p* = 0.7323), 25 *μ*g/mL (61.71*%* ± 2.42*%*, *p* = 0.9465), and 50 *μ*g/mL (61.02*%* ± 3.17*%*, *p* = 0.8762), these did not reach statistical significance. At higher concentration (100 *μ*g/mL), there was a significant decrease in cell viability (Figure 4), and therefore, glucose utilization and uptake at this concentration should be interpreted with caution.

### 3.7. INS‐1 Pancreatic *β*‐Cell Proliferation Assay

**Figure 7:**
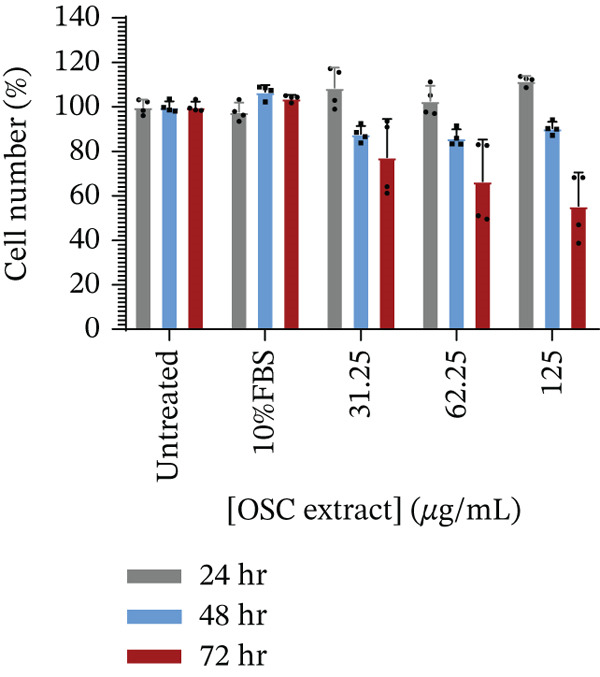
Effect of the sample on INS‐1 pancreatic *β*‐cell proliferation.

The potential for the test sample to promote pancreatic *β*‐cell growth was assessed as described in a previous study [41]. INS‐1 *β*‐cells were exposed to three concentrations of the sample (31.25, 62.5, and 125 *μ*g/mL), alongside untreated controls and a positive control (10% FBS), and monitored at 24, 48, and 72 h. After 24 h, all tested concentrations produced modest increases in cell counts relative to the untreated control: 31.25 *μ*g/mL (108.66*%* ± 9.05*%*, *p* = 0.0712), 62.5 *μ*g/mL (102.76*%* ± 6.74*%*, *p* = 0.2539), and 125 *μ*g/mL (111.79*%* ± 2.18*%*, *p* = 0.0001). Although statistically significant at the highest concentration, the magnitude of this increase was relatively small and only slightly higher than that observed with the positive control (10% FBS; 97.78*%* ± 4.14*%*). At 48 and 72 h, however, cell numbers declined with increasing concentration and exposure time. These reductions were not statistically significant (*p* > 0.05) but indicated a downward trend compared with the 24‐h time point. In contrast, the positive control (10% FBS) maintained higher cell counts at both later time points (Figure 7).

## 4. Discussion

While previous animal studies show that *Opuntia* spp. can lower blood sugar levels, its exact mechanisms of action are unclear. Hyperglycemia is the major symptom of T2DM and is due to the absence of insulin or resistance to insulin. Therefore, the current study on Zambian‐grown OSC assessed the targeted mechanisms that control blood glucose levels, particularly postprandial glucose control, pancreatic *β*‐cell proliferation, and glucose uptake in L6 myoblasts and C3A hepatocytes.

The solvent‐dependent variation in phytochemical extraction observed in this study is consistent with previous reports on *Opuntia* species. Methanol exhibited the highest extraction efficiency, yielding a broad spectrum of phytochemicals including reducing sugars, alkaloids, tannins, glycosides, and carbohydrates, which supports earlier findings that highly polar solvents are effective in recovering diverse polar secondary metabolites from cactus cladodes [42, 43]. In contrast, studies on *Opuntia stricta* and *Opuntia ficus-indica* have reported higher phenolic and flavonoid yields using acetone‐rich solvent systems, particularly aqueous acetone [44, 45]. Consistent with solvent polarity principles, acetone in the present study was comparatively more efficient in extracting triterpenoids and sterols, compounds typically associated with lower polarity solvents [44, 45]. The absence of anthraquinones across all solvent extracts also agrees with previous phytochemical characterizations of OSCs [42].

Notably, several phytochemicals identified in this study, including alkaloids, tannins, glycosides, and carbohydrates, have been widely reported to exert antidiabetic effects through mechanisms such as enhancement of insulin secretion, increased glucose uptake, inhibition of carbohydrate‐digesting enzymes, antioxidant protection of pancreatic *β*‐cells, and delayed intestinal glucose absorption [46–48]. These findings therefore reinforce the potential of OSCs as a promising source of antidiabetic bioactive compounds.

Assessment of probable toxicity is a key first step before undertaking in vitro studies, as it safeguards experimental models and guarantees the reliability of results [38, 41]. When the safety is assured, evaluation of the antidiabetic pharmacological activity of test compounds ensures incorporation of traditional medicines into public health programs [26, 38]. The OSC methanol extract showed favorable tolerability across all three tested cell lines. In L6 myoblasts, no cytotoxicity was observed at any concentration, with viability consistently above 90%. In C3A hepatocytes and Caco‐2 epithelial cells, viability declined only at the highest concentration (100 mg) but remained above the 80% noncytotoxicity threshold defined by ISO 10993‐5:2009. These results align with reports from South Korea and Morocco, where *Opuntia ficus-indica* var. *saboten* cladodes and *Opuntia dillenii* seed oil were also nontoxic to L6 myoblasts and HepG2 cells, respectively [18, 24, 25]. Similarly, a study conducted in Tunisia demonstrated that polysaccharides isolated from OSC did not induce cytotoxicity in HepG2 cells. These observations suggest that different species and plant parts of *Opuntia* exhibit a favorable safety profile across distinct cellular models and support the potential application of OSC extracts in pharmacological and nutraceutical development.

In this study, glucose utilization in C3A hepatocytes increased significantly in a dose‐dependent manner across all tested concentrations. This observation indicates that the OSC extract did not impair the metabolic activity of C3A cells. However, comparison with other studies is limited due to the scarcity of published reports describing similar findings. In our study, glucose uptake in C3A hepatocytes also increased, with the peak effect observed at the highest concentration, with responses not significantly different from those observed in insulin‐treated cells under the experimental conditions. This pattern suggests that the compound may facilitate hepatic glucose handling by promoting glycogen synthesis or enhancing GLUT2‐mediated uptake. Given the liver′s central role in regulating postabsorptive glucose levels, these results point to potential direct effects on hepatocyte metabolic pathways. Mechanistically, the compound could be activating AMP‐activated protein kinase (AMPK) or modulating insulin receptor substrates, leading to improved hepatic glucose clearance. To the best of our knowledge, no published study has evaluated the effect of OSC extracts on glucose uptake in C3A hepatocytes at the time this study was conducted.

In L6 myoblasts, glucose utilization and uptake were evaluated in the present study. Glucose utilization, assessed after 24‐h exposure to OSC extract, increased significantly only at 100 *μ*g/mL versus control (95.1% vs. 93.5%, *p* < 0.005), with no differences at 6.25–50 *μ*g/mL. This indicates that OSC did not impair myocellular metabolic activity across the tested range and produced a small enhancement at the top dose. Glucose uptake showed a modest, concentration‐related rise, reaching significance only at 100 *μ*g/mL versus control (53.7% vs. 48.6%). The 6.25–25 *μ*g/mL doses were indistinguishable from control, and 50 *μ*g/mL produced a small, nonsignificant increase. The insulin positive control produced a higher response than the lower OSC concentrations, while the effect observed at 100 *μ*g/mL was not significantly different from that of insulin (54.3% vs. 53.7%). This suggests that OSC at the highest tested concentration elicited a response of similar magnitude under the experimental conditions. These findings align with prior work from South Korea showing that *Opuntia ficus-indica* var. *saboten* enhances glucose uptake in L6 cells [18]. These findings suggest that *Opuntia* spp. possess insulin‐sensitizing activity, plausibly mediated via activation of AMPK, a central regulator of glucose transport in skeletal muscle [49, 50].

In the current study, Caco‐2 cells showed no significant differences in glucose utilization across all tested concentrations, likely due to near‐maximal glucose depletion under baseline conditions. Under these conditions, cells had already consumed most of the available glucose during the incubation period, thereby limiting the sensitivity of the model to detect treatment‐related differences. In contrast, glucose uptake was significantly reduced at 6.25 and 100 *μ*g/mL; however, the effect observed at 100 *μ*g/mL was confounded by reduced cell viability and should therefore be interpreted with caution. Notably, the reduction at 6.25 *μ*g/mL in viable cells suggests a potential inhibitory effect on intestinal glucose uptake. However, the absence of significant effects at intermediate concentrations (12.5–50 *μ*g/mL) indicates that this observation may be concentration‐dependent and should be considered preliminary rather than definitive. While this effect may involve modulation of intestinal glucose transporters such as SGLT1 or GLUT2, no direct assessment of transporter expression or activity was performed in the present study, and thus, these interpretations remain speculative. Future studies incorporating transporter expression analyses, cotreatment with specific inhibitors, and radiolabeled glucose uptake assays are warranted to provide stronger biological and pathway‐based validation of these findings. Collectively, these results suggest that OSC may exert a potential, yet preliminary, effect on intestinal glucose handling, which could complement its insulin‐mimetic activity observed in myocytes and hepatocytes. However, further validation of the underlying biological processes is required to substantiate its role in modulating postprandial glucose dynamics.

In the present study, OSC produced a modest increase in INS‐1 *β*‐cell numbers after 24 h, with the highest concentration (125 *μ*g/mL) showing a statistically significant increase relative to the untreated control. However, the magnitude of this increase was small and only slightly higher than that observed with the 10% FBS positive control, and therefore, its biological relevance should be interpreted with caution. In addition, the assay relied on total cell counts, which reflect the net balance between proliferation, survival, and cell death, and cannot conclusively confirm true proliferative activity. By 48 and 72 h, cell numbers declined with increasing concentration and exposure time, indicating a reduction in cell growth following prolonged exposure to the extract. However, because apoptosis or cell death markers were not assessed in this study, the underlying mechanism responsible for this decline cannot be conclusively determined. In contrast, 10% FBS maintained higher cell numbers across all time points, indicating that the observed effect of the extract was transient. Nevertheless, these findings are consistent with previous studies reporting beneficial effects of *Opuntia* species on pancreatic *β*‐cell function. Extracts from *Opuntia* species have been shown to increase *β*‐cell numbers and improve pancreatic islet function in db/db mice [18, 51], while polysaccharides from *Opuntia milpa alta* protected INS‐1 cells from alloxan‐induced apoptosis [52]. Collectively, these findings suggest that *Opuntia* species may support *β*‐cell viability and function, although further studies using specific proliferation markers and complementary assays are needed to clarify the underlying mechanisms.

## 5. Strengths and Limitations of the Study

The present study provides one of the first functional investigations of Zambian‐grown OSC extracts across multiple glucose‐regulating pathways using complementary cellular models, including C3A hepatocytes, L6 myoblasts, Caco‐2 intestinal epithelial cells, and INS‐1 pancreatic *β*‐cells. This multimodel approach enabled the assessment of potential effects on intestinal glucose absorption, peripheral glucose utilization, hepatic glucose handling, and *β*‐cell dynamics within a single experimental framework. Additionally, cytotoxicity was evaluated prior to functional assays to ensure that the observed metabolic responses were not attributable to overt cellular toxicity. However, several limitations should be acknowledged. The study utilized a crude methanolic extract, and therefore, the specific bioactive compounds and their molar concentrations were not characterized. The oral bioavailability and pharmacokinetic behavior of the active constituents were also not assessed, limiting extrapolation to physiological conditions. Furthermore, the increases in glucose uptake observed in vitro were modest, and their clinical relevance remains uncertain. The *β*‐cell assay relied on total cell counts rather than specific proliferation markers and, therefore, cannot conclusively distinguish between proliferation, enhanced survival, or reduced cell death. Finally, the proposed pathways involving processes such as AMPK activation or glucose transporter modulation were not directly measured, and the findings are limited to in vitro models, necessitating further detailed and in vivo investigations.

## 6. Conclusion

This study provides the first comprehensive evidence that OSC extracts exert multifaceted antidiabetic actions. Specifically, OSC enhanced glucose utilization and uptake in C3A hepatocytes and L6 skeletal myoblasts to levels similar to those observed in insulin‐treated cells under the experimental conditions, suggesting a potential insulin‐mimetic effect in peripheral tissues. The extracts also significantly reduced intestinal glucose absorption in Caco‐2 cells, suggesting a capacity to blunt postprandial glycemic excursions. Moreover, the observed transient increase in pancreatic *β*‐cell numbers beyond that seen with 10% FBS suggests a modest effect that may be indicative of potential *β*‐cell preservation or supportive cellular responses. Together, these findings highlight OSC as a novel source of bioactive compounds capable of targeting multiple pathophysiological mechanisms of diabetes, including insulin resistance, postprandial hyperglycemia, and *β*‐cell decline. These results establish a strong rationale for subsequent molecular pathway investigations, bioactive compound isolation, and rigorous in vivo evaluation to validate OSC′s translational potential as a phytotherapeutic candidate for diabetes management.

## Author Contributions

M.K., A.G.B., and C.C.E. contributed to designing the research. A.G.B. and C.C.E. supervised and coordinated and were responsible for quality control. M.K. and K.Z. contributed to material preparation and data collection. M.K. analyzed the data. M.K., A.G.B., and C.C.E. contributed to the interpretation of the analyses of data. The first draft of the manuscript was written by M.K., A.G.B., C.C.E., and K.Z.

## Funding

No funding was received for this manuscript.

## Disclosure

M.K, the lead author, declares that this article is an honest, accurate, and transparent account of the study that is being reported; and that no significant elements of the investigation have been omitted. All authors commented on the previous versions of the manuscript and have read and approved the final version of the manuscript.

## Ethics Statement

The protocol for this study was approved by the University of Zambia School of Health Sciences Research Ethics Committee (Study protocol ID: 202112030063). We obtained informed, written consent from all participants before the study. The participants′ confidentiality and anonymity were strictly maintained.

## Consent

Informed consent from all the participants was taken.

## Conflicts of Interest

The authors declare no conflicts of interest.

## Data Availability

Upon reasonable request, the corresponding author, M.K., would provide the data that support the study′s conclusions.
